# Reduced Cardiac Index Reserve and Hypovolemia in Severe Falciparum Malaria

**DOI:** 10.1093/infdis/jiz568

**Published:** 2019-11-06

**Authors:** Hugh W F Kingston, Aniruddha Ghose, Voravut Rungpradubvong, Sudarat Satitthummanid, M Trent Herdman, Katherine Plewes, Stije J Leopold, Haruhiko Ishioka, Sanjib Mohanty, Richard J Maude, Marcus J Schultz, Wim K Lagrand, Md Amir Hossain, Nicholas P J Day, Nicholas J White, Nicholas M Anstey, Arjen M Dondorp

**Affiliations:** 1 Global and Tropical Health Division, Menzies School of Health Research and Charles Darwin University, Darwin, Australia; 2 Mahidol Oxford Tropical Medicine Research Unit, Faculty of Tropical Medicine, Mahidol University, Bangkok, Thailand; 3 Chittagong Medical College, Chittagong, Bangladesh; 4 Centre for Tropical Medicine and Global Health, Nuffield Department of Clinical Medicine, Churchill Hospital, Oxford, United Kingdom; 5 Division of Cardiology, Department of Medicine, Faculty of Medicine, Chulalongkorn University, Bangkok, Thailand; 6 Cardiac Center, King Chulalongkorn Memorial Hospital, Bangkok, Thailand; 7 ISPAT General Hospital, Rourkela, Orissa, India; 8 Department of Intensive Care Medicine, Academic Medical Center, University of Amsterdam, The Netherlands

**Keywords:** echocardiography, hemodynamics, hypovolemia, severe malaria, systolic function

## Abstract

**Background:**

Impaired microvascular perfusion is central to the development of coma and lactic acidosis in severe falciparum malaria. Refractory hypotension is rare on admission but develops frequently in fatal cases. We assessed cardiac function and volume status in severe falciparum malaria and its prognostic significance.

**Methods:**

Patients with severe (N = 101) or acute uncomplicated falciparum malaria (N = 83) were recruited from 2 hospitals in India and Bangladesh, and healthy participants (N = 44) underwent echocardiography.

**Results:**

Patients with severe malaria had 38% shorter left ventricular (LV) filling times and 25% shorter LV ejection times than healthy participants because of tachycardia; however, stroke volume, LV internal diameter in diastole (LVIDd), and LV internal diameter in systole (LVIDs) indices were similar. A low endocardial fraction shortening (eFS) was present in 17% (9 of 52) of severe malaria patients. Adjusting for preload and afterload, eFS was similar in health and severe malaria. Fatal cases had smaller baseline LVIDd and LVIDs indices, more collapsible inferior vena cavae (IVC), and higher heart rates than survivors. The LVIDs and IVC collapsibility were independent predictors for mortality, together with base excess and Glasgow Coma Scale.

**Conclusions:**

Patients with severe malaria have rapid ejection of a normal stroke volume. Fatal cases had features of relative hypovolemia and reduced cardiac index reserve.

## Teaser

Echocardiography in a cohort of patients with severe malaria showed that systolic and diastolic dysfunction was infrequent but that patients who died had evidence of hypovolemia and reduced cardiac index reserve.

Severe falciparum malaria caused an estimated 435 000 deaths globally in 2017 [1]. Vital organ dysfunction develops in patients with high parasite biomass infections [2]. Reduced microcirculatory perfusion resulting from microvascular obstruction by sequestered parasites is central to the development of coma and lactic acidosis [3, 4]. Although dehydration is common and hypovolemia may occur [5–8], large volume fluid resuscitation is not associated with resolution of acidosis [6] and may result in pulmonary edema [6]. Rapid administration of large fluid volumes is associated with secondary hypotension and increased mortality in children [9]. These findings suggest that microvascular obstruction and dysfunction (rather than hypovolemia or insufficient cardiac output) lead to inadequate tissue perfusion [4, 10].

The causal chain of events resulting in death from severe malaria has multiple links. The factors that cause severe disease to develop (including sequestration of a high parasite biomass infection) are not necessarily equivalent to the factors affecting subsequent survival. Although hypovolemia does not cause coma or acidosis, macrovascular stability could determine survival after the start of treatment because refractory hypotension is a common terminal event in patients with severe malaria [11]. The cardiovascular system in malaria is hyperdynamic, with an increase in the ratio of oxygen consumption to delivery resulting from increased oxygen consumption and anemia [12]. There have been few studies of cardiac function in adult patients with severe malaria [13, 14]. Post-mortem studies of the heart in adults and children dying from severe falciparum malaria have shown that sequestration of parasitized erythrocytes in the cardiac microvasculature is common [15, 16].

We studied the relationships between disease severity, cardiac function, and volume status in malaria. We hypothesized that reduced cardiac index reserve resulting from hypovolemia and impaired systolic function are associated with increased mortality.

## METHODS

### Patients

Consecutive consenting patients admitted to the adult medical wards during the malaria seasons at Chittagong Medical College Hospital (CMCH), Chittagong, Bangladesh in 2011–2014 and ISPAT General Hospital (IGH), Rourkela, India in 2011 with severe or uncomplicated falciparum malaria as defined previously [17] were enrolled into a pathophysiology study. There was no age cutoff, and adolescents admitted to the adult wards were also included. Patients with severe pre-existing cardiovascular disease were excluded from this analysis. Healthy subjects with no known acute or chronic illnesses were recruited locally. Further details can be found in the Supplementary Methods. Written informed consent was obtained from all participants or, for patients lacking capacity to provide consent, their representatives, or those who were <18 years old. The study was approved by the Oxford Tropical Research, Chittagong Medical College and IGH ethical committees. All patients had a medical history taken, full clinical examination, blood draw, and echocardiography performed on enrollment and were followed up at least once every 6 hours. Between 2011 and 2012, a focused echocardiographic exam measuring only cardiac index and inferior vena cava (IVC) diameters was conducted, whereas from 2013 to 2014 a detailed exam was completed. Full details of echocardiographic procedures are provided in the Supplementary Methods.

### Statistics

Groups were compared using Mann-Whitney *U* tests (2 groups) or Kruskal-Wallis tests (more than 2 groups). Correlations were assessed using Spearman’s rank. Linear regression was used to adjust indices of ventricular function for preload, afterload, and ejection time [18]. Within the group of patients with severe malaria, the prognostic significance of variables was assessed by calculation of the area under the receiver operator curve (AUROC). Multivariate logistic regression was used to assess whether echo variables that were significant univariate predictors of mortality were independent predictors of mortality alongside the established predictors; Glasgow Coma Score (GCS) and base excess. All analyses were conducted using Stata, version 15 (StataCorp, College Station, TX).

## RESULTS

### Baseline Characteristics and Recruitment

Data from detailed and focused echocardiographic examinations, respectively, were available from 52 and 49 patients with severe malaria, 30 and 53 patients with uncomplicated malaria, and 32 and 12 healthy participants, respectively. Of these patients, 5 with uncomplicated malaria and 3 with severe malaria had a history of cardiovascular disease (hypertension only). The healthy control group had larger body surface areas than the malaria groups (both *P* < .001) (Table 1). The age range in the severe malaria group was from 10- to 70-year-olds with 8% (8 of 101) being under 18 years. Two patients with severe malaria were receiving inotropes/vasopressors, and 1 patient was mechanically ventilated around the time of assessment. Mortality was 34% (34 of 101) in severe malaria. The volumes of intravenous fluid patients with severe malaria had received before enrollment were similar in those who died (22 mL/kg; interquartile range [IQR], 11–41; N = 20) and in those who survived (22 mL/kg; IQR,11–41; N = 43) (*P* = .895).

**Table 1.** Baseline Characteristics^a^

Variable	Healthy (N = 44)	Uncomplicated (N = 83)	Severe (N = 101)	Severe—Alive (N = 67)	Severe—Dead (N = 34)	Overall	UM vs SM	Alive vs Dead
Age (years)	27 (24 to 35)	26 (20 to 40)	30 (24 to 42)	30 (22 to 40)	30 (24 to 45)	0.371	0.201	0.903
Sex (%male)	82%	71%	69%	75%	59%	0.286	0.793	0.104
BSA (m^2^)	1.69 (1.55 to 1.8)	1.55 (1.38 to 1.68)	1.53 (1.46 to 1.66)	1.52 (1.43 to 1.66)	1.55 (1.48 to 1.66)	<0.001	0.906	0.39
Temperature (°C)	36.7 (36.5 to 37)	37.6 (37 to 38.8)	38.1 (37.2 to 38.9)	37.9 (37.2 to 38.9)	38.4 (37.2 to 39.1)	<0.001	0.133	0.595
SBP (mmHg)	121 (113 to 131)	108 (99 to 116)	110 (101 to 121)	109 (100 to 120)	114 (103 to 129)	<0.001	0.147	0.233
DBP (mmHg)	72 (66 to 82)	62 (54 to 69)	65 (57 to 76)	65 (58 to 75)	65 (56 to 79)	<0.001	0.099	0.846
MAP (mmHg)	90 (82 to 97)	78 (71 to 85)	81 (72 to 91)	81 (72 to 91)	82 (71 to 95)	<0.001	0.075	0.419
HR (/minute)	77 (69 to 84)	100 (88 to 112)	113 (95 to 128)	104 (91 to 122)	124 (108 to 134)	<0.001	<0.001	<0.001
SaO_2_ (%)	97 (96 to 98)	97 (96 to 98)	96 (94 to 97)	96 (95 to 97)	96 (93 to 97)	<0.001	<0.001	0.107
Hematocrit (%)	44 (38 to 46)	30 (24 to 36)	26 (21 to 32)	27 (22 to 33)	25 (19 to 32)	<0.001	0.007	0.24
Parasitemia (/µL)	NA	13 138 (1200 to 55 465)	98 872 (16 768 to 296 956)	74 732 (12 208 to 284 107)	135 114 (21 101 to 401 631)	<0.001	<0.001	0.534
BE (mmol)	NA	−1 (−3 to 0)	−8 (−11 to −4)	−6 (−10 to −4)	−11 (−16 to −7)	<0.001	<0.001	<0.001
Creatinine (mg/dL)	NA	1 (0.8 to 1.2)	1.42 (1.02 to 3.54)	1.37 (1 to 3.53)	1.57 (1.03 to 4.23)	<0.001	<0.001	0.407
Lactate (mmol/L)	NA	1.5 (1.2 to 2)	3.8 (2.5 to 6.3)	2.9 (2 to 4.7)	6.2 (3.9 to 9.9)	<0.001	<0.001	<0.001
Coma (%GCS <11)	NA	0%	65%	58%	79%	<0.001	<0.001	0.034

Abbreviations: BE, base excess; BSA, body surface area; DBP, diastolic blood pressure; GCS, Glasgow coma score; Hct, hematocrit; HR, heart rate; MAP, mean arterial pressure; NA, not applicable; NR, not recorded; SaO_2_, arterial oxygen saturation; SBP, systolic blood pressure; SM, severe malaria; UM, uncomplicated malaria.

^a^Shown is median (interquartile range) for nonbinary data, or percentage for binary data. Overall *P* value is from Kruskal-Wallis test comparing groups of healthy participant, severe and uncomplicated malaria.

### Heart Rate, Stroke Volume, and Cardiac Output

**Figure 1.**
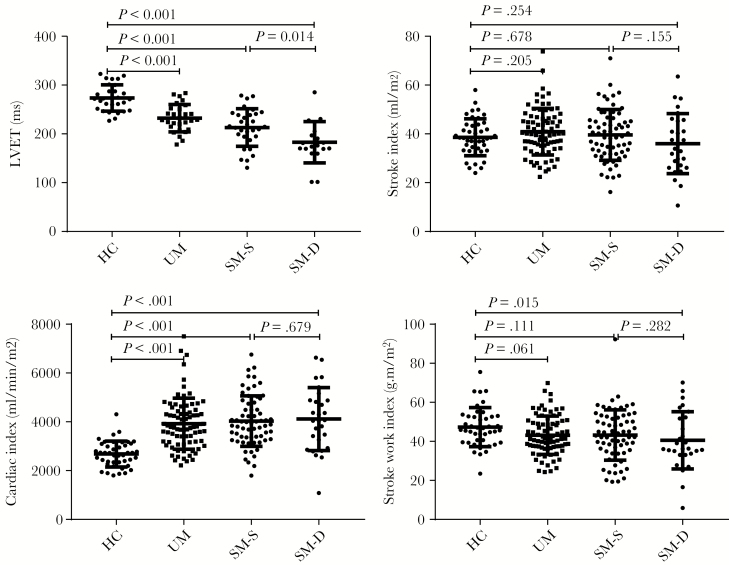
Left ventricular ejection time, stroke and cardiac index and stroke work index. Bars show median and interquartile range. HC, hematocrit; LVET, left ventricular ejection time; SM, severe malaria; UM, uncomplicated malaria.

Despite the increases in heart rate with increasing malaria severity (*P* < .001 test for trend) (Table 1), and the corresponding reductions in ejection time (Figure 1A), stroke index values were similar in severe and uncomplicated malaria patients compared with healthy participants (Figure 1B). Therefore, cardiac index (Figure 1C) was increased in malaria in proportion to the rise in heart rate. Values of stroke work index, a measure of the external work done by the heart, were mildly reduced in malaria relative to healthy persons (Figure 1D, Supplementary Table 1), whereas values of stroke power index, a measure of the rate at which work is being done by the heart, increased modestly in malaria as the shortening in ejection time outweighed the decrease in mean arterial pressure in malaria (Supplementary Table 1). Cardiac power index values were increased similarly by approximately 35% in severe malaria relative to health because of the higher heart rates (Supplementary Table 1). Fatal cases had higher heart rates (AUROC, 0.71; 95% confidence interval [CI], 0.6–0.81) (Table 1) than survivors.

### Cardiac Chamber Dimensions

**Figure 2.**
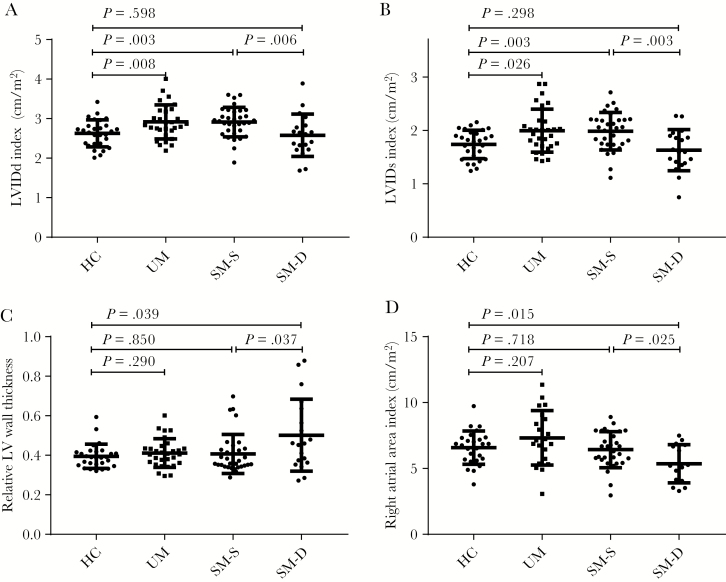
Chamber volumes. Bars show median and interquartile range. HC, hematocrit; LV, left ventricular; LVIDd, LV internal diameter in diastole; LVIDs, LV internal diameter in systole; SM, severe malaria; UM, uncomplicated malaria.

**Figure 3.**
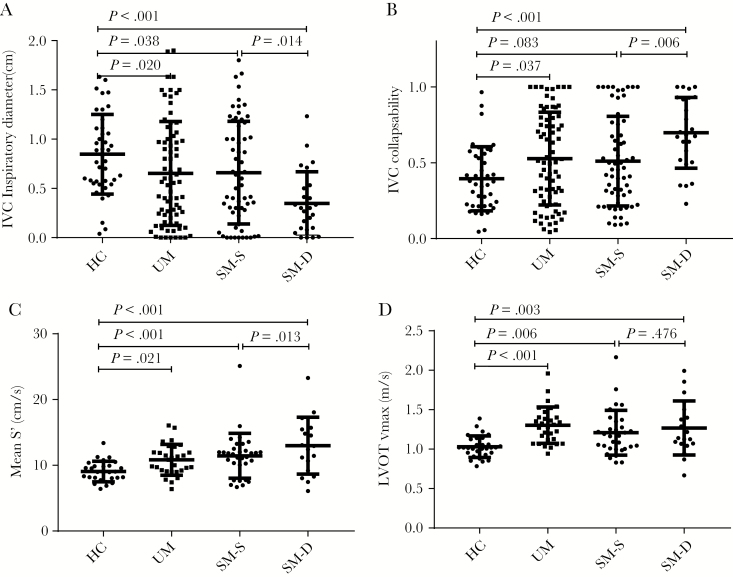
Inferior vena cava (IVC) diameter and collapsibility, S’ and left ventricular outflow tract maximum velocity (LVOT_vmax_). Bars show median and interquartile range. HC, hematocrit; SM, severe malaria; UM, uncomplicated malaria.

Median chamber dimensions in patients with uncomplicated malaria were mostly within the normal range but were larger than in healthy participants (Figure 2, Supplementary Table 2). Patients with severe malaria tended to have smaller cardiac dimensions than in uncomplicated malaria, similar to healthy participants (Figure 2, Supplementary Table 2). The IVC inspiratory diameter was smaller in malaria, and IVC collapsibility, a predictor of fluid responsiveness, was greater (Figure 3A and B). The IVC collapsibility did not correlate significantly with respiratory rate in severe malaria (rho = 0.17, N = 85, *P* = .170) and was not associated with dyspnea in the severe malaria group (*P* = .10).

Patients who died had smaller left ventricular (LV) internal diameters in diastole (LVIDd) (AUROC, 0.73; 95% CI, 0.57–0.89) (Figure 2A) and LV internal diameters in systole (LVIDs) (AUROC, 0.75; 95% CI, 0.61–0.89) (Figure 2B) and larger relative wall thicknesses (AUROC, 0.67; 95% CI, 0.51–0.84) (Figure 2C) than those who survived, despite similar estimated LV mass (Supplementary Table 2). The right atrial (RA) area index (AUROC, 0.71; 95% CI, 0.54–0.88) (Figure 2D) and IVC inspiratory diameter (AUROC, 0.67; 95% CI, 0.55–0.79) (Figures 3A) values were both smaller fatal cases, whereas IVC collapsibility (AUROC, 0.69; 95% CI, 0.58–0.8) was increased in fatal cases (Figures 3B).

### Systolic Function

In severe malaria values for average systolic mitral annular tissue velocity (S’) (Figure 3C) and maximal LV outflow tract velocity (LVOT_Vmax_) (Figure 3D), both measures of peak LV contraction rate were increased, whereas stroke index values were similar relative to healthy participants (Figure 1B). Increased peak ejection velocity was associated with a reduction in afterload in severe malaria, with average mitral annular S’ correlating inversely with mediorotational end systolic stress (MRESS) (rho = −0.47, N = 51, *P* ≤ .001) and systemic vascular resistance index (SVRI) (rho = −0.42, N = 47, *P* = .004).

**Figure 4.**
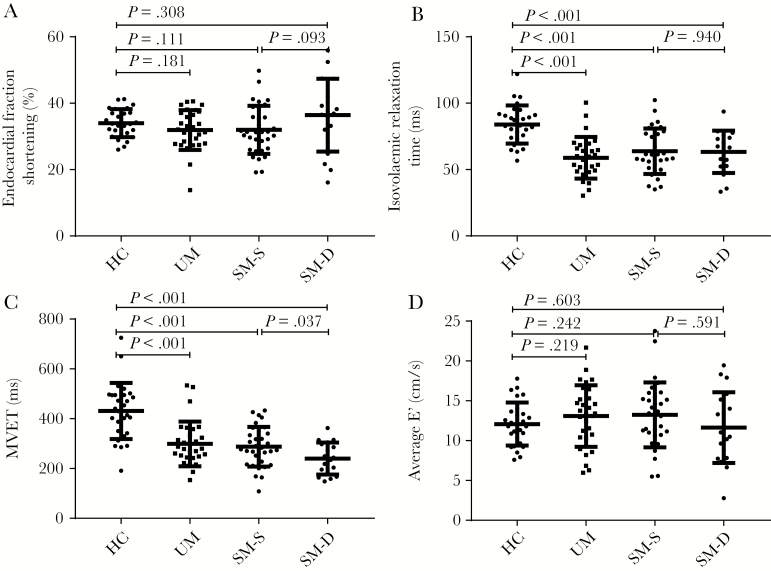
Left ventricular endocardial fraction shortening and diastolic function indices. Bars show median and interquartile range. HC, hematocrit; MVET, mitral valve ejection time; SM, severe malaria; UM, uncomplicated malaria.

Endocardial fractional shortening (eFS) (Figure 4A) values were low (<25%) in 7% (2 of 30) of patients and high (>45%) in none of the patients with uncomplicated malaria, whereas in severe malaria, eFS values were low in 17% (9 of 52) of patients and high in 13% (7 of 52). In severe malaria, eFS was not associated with mortality when analyzed as a continuous variable (Supplementary Table 3), and having a low eFS was not associated with increased mortality (nonlow eFS 15 of 43 [35%] versus low eFS 4 of 9 [44%]; *P* = 0.58), However, high eFS values were associated with a higher risk of a fatal outcome (14 of 45 [31%] versus 5 of 7 [71%]; *P* = 0.039). Values of tricuspid annular plane systolic excursion, as a measure of right ventricular (RV) systolic function, were low (<1.6 cm) in 7% (2 of 29) of uncomplicated and 12% (6 of 49) of severe malaria cases and high (>3 cm) in 3% (1 of 29) of uncomplicated and 6% (3 of 49) of severe malaria cases.

**Figure 5.**
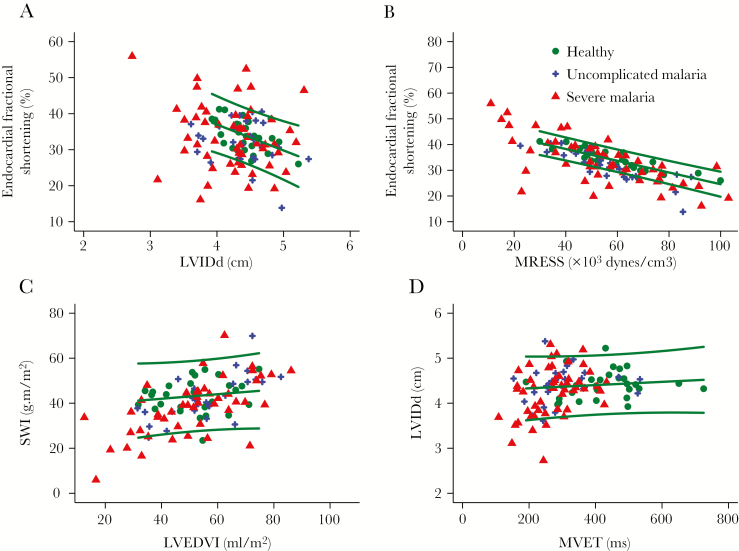
(A) Relationship between left ventricular internal diameter in diastole (LVIDd) and endocardial fractional shortening. (B) Relationship between mediorotational end systolic stress (MRESS) and endocardial fractional shortening. (C) Left ventricular end diastolic volume index (LVEDVI)—stroke work relationship. (D) Mitral valve ejection time (MVET)—LVIDd relationship. Lines indicate mean and 95% prediction interval for healthy persons.

Left ventricular systolic performance was also assessed in relation to preload (LVIDd) (Figure 5A) and afterload (MRESS) (Figure 5B). Patients with severe malaria performed both above and below the prediction interval derived from the healthy participant data. In a linear regression model for eFS using LVIDd, MRESS, sex (a known determinant of eFS), and participant category (Supplementary Table 4), eFS was similar in severe malaria to healthy persons but lower in uncomplicated malaria than healthy persons. When stroke work index was considered (Figure 5C), after adjusting for LV end diastolic volume index (LVEDVI) and sex, stroke work index was similar in uncomplicated and lower in severe malaria than in healthy persons (Supplementary Table 4). Neither stroke work index nor eFS take work rate into account, whereas stroke power does; when stroke power index was considered, after adjusting for LVEDVI and gender, stroke power index values were similar in uncomplicated malaria and higher in severe malaria than healthy participants (Supplementary Table 4).

### Left Ventricular Diastolic Function

With the exceptions of the isovolemic relaxation time ([IVRT] a measure of the rate at which the ventricle relaxes) (Figure 4B) and mitral valve ejection time (MVET) (Figure 4C), which were both low in severe and uncomplicated malaria, variables related to diastole including E’ (a measure of LV relaxation rate) (Figure 4D) were relatively unchanged by malaria and did not predict mortality (Supplementary Table 5). A low E’ (septal <7 or lateral <10 cm/s) was present in 3 of 28 (10%) healthy persons, 5 of 30 (17%) of patients with uncomplicated malaria, and 13 of 51 (25%) of patients with severe malaria (*P* = .23). The relationship between filling time and LVIDd is shown in Figure 5D.

In severe malaria, LVIDd was correlated positively with average mitral peak early diastolic tissue velocity (E’) (rho = 0.37, N = 51, *P* = .007), MVET (rho = 0.37, N = 51, *P* = .007), but not early mitral peak E-wave velocity (E), IVRT, or IVC diameter or collapsibility (all *P* > .05), suggesting that in the severe malaria group, filling time and possibly relaxation are the main determinants of LVIDd. The IVRT and average E’ were inversely associated (rho = −0.6, N = 47, *P* < .001). The LVIDs was not associated with E, average E’, or IVRT (all *P* > .05).

### Relationship Between Age, Anemia, Fever, Heart Rate, Acidosis, and Echocardiographic Measures in Severe Malaria

In patients with severe malaria, age was not associated with LVIDd or LVIDs (both *P* > .05) but was negatively associated with average E’ (rho = −0.50, N = 51, *P* < .001) and E (rho = −0.63, N = 52, *P* < .001) and was positively associated with IVSd (rho = 0.46, N = 52, *P* < .001), relative LV wall thickness (RWT) (rho = 0.37, N = 52, *P* = .007) and IVRT (rho = 0.46, N = 48, *P* = .001), consistent with the known age-related transition to concentric geometry and reduced relaxation.

Tachycardia was not associated with hematocrit (rho = −0.24, N = 52, *P* = .094), but it was positively correlated with temperature (rho = 0.42, N = 52, *P* = .002). Tachycardia was associated with smaller LVIDd (rho = −0.46, N = 47, *P* = .001), LVIDs (rho = −0.56, N = 47, *P* < .001), and stroke index values (rho = −0.39, N = 47, *P* = .006) and larger medial S’ (rho = 0.48, N = 47, *P* < .001) and eFS (rho = 0.33, N = 47, *P* = .022) values but not E or average E’ (both *P* > .05).

Hematocrit was inversely correlated with cardiac index (rho = −0.51, N = 47, *P* < .001), stroke index (rho = −0.41, N = 47, *P* = .004), and eFS (rho = −0.37, N = 52, *P* = .007). In a univariate analysis, markers of volume status or filling pressure were not associated with hematocrit; there was no correlation with LVIDd or IVC dimensions or E/E’ (*P* > .05). Hematocrit correlated inversely with the systolic function parameters average S’ (rho = −0.36, N = 51, *P* = .009) and LVOT_Vmax_ (rho = −0.50, N = 52, *P* < .001). Hematocrit was also associated positively with the diastolic parameter IVRT (rho = 0.42, N = 48, *P* = .026) and inversely with E (rho = −0.46, N = 52, *P* < .001) and E’ (rho = −0.38, N = 51, *P* = .007). Examining the associations with afterload indices, hematocrit correlated with SVRI (rho = 0.46, N = 47, *P* = .001) but not MRESS or mean arterial pressure (*P* > .05). Linear regression was used to explore whether the larger eFS in anemia was related to an increase in contractility or loading conditions. Consistent with hematocrit increasing eFS through changes in loading as opposed to contractility, in a linear regression model for eFS, after adjusting for sex, MRESS and LVIDd hematocrit was not a significant predictor (likelihood ratio test, *P* = .752). In a linear regression model for LVIDd, adjusted for gender and MVET, hematocrit was a positive predictor, suggesting that preload is higher in anemia.

Venous plasma lactate concentrations were positively associated with heart rate (rho = 0.38, N = 108, *P* < .001) and eFS (rho = 0.33, N = 47, *P* = .021) and negatively associated with LVIDd (rho = −0.36, N = 47, *P* = .012) and LVIDs (rho = −0.46, N = 47, *P* = .001) but not stroke index, mean S’, IVRT, or IVC dimensions, fever, or hematocrit (*P* > .05).

### Multivariate Prediction of Mortality

The predictors of mortality in the univariate analysis were combined with the previously established predictors of mortality, base excess, and GCS, to assess whether macrovascular parameters were independent prognostic indicators. The RA area index, relative wall thicknesses, and LVIDd index were not significant independent predictors (*P* > .05), whereast heart rate, IVC collapsibility, IVC inspiratory diameter, and LVIDs index were significantly associated with a fatal outcome independent of base excess and GCS (Supplementary Table 6).

## DISCUSSION

Macrovascular cardiovascular parameters predicted outcome in this cohort of patients despite the fact that coma and lactic acidosis in severe falciparum malaria was not caused by inadequate cardiac output or total oxygen delivery. Differences in cardiac function parameters between severe and uncomplicated malaria were small (smaller atria, shorter ejection time, and higher heart rate in severe disease), but more substantial changes separated severe malaria survivors from fatal cases. Patients who died had higher heart rates, with smaller LV and RA diameters, increased IVC collapsibility, and high LV fractional shortening. Patients presenting with these cardiovascular profiles are expected to have lower cardiac index reserve and hence might be less able to compensate further peripheral vasodilation or other hemodynamic stresses. There was no evidence that systolic LV or RV dysfunction predicted outcome.

Previous studies of hemodynamics in severe malaria have lacked power to assess the association between macrovascular hemodynamic parameters and mortality. In severe malaria, no clinically significant acute change in LVIDd was noted in 2 studies in children [8, 19] and 1 study in adults [13], and baseline eFS or ejection fraction was usually normal [8, 10, 13, 14, 19, 20]. In children, insulin sensitivity (SI) decreased on recovery in one study in which there was a corresponding increase in hematocrit [19] and increased in another [8]. An increase in IVC collapsibility has been reported in children [8]. One study in children found evidence of increased tricuspid regurgitant jet velocity, suggesting that pulmonary hypertension may occur [20]. Impaired cardiac function in falciparum malaria is rare, presenting as myocarditis, reduced ejection fractional, or acute coronary syndrome in children [21] and adults [22–25].

In this study, malaria was associated with an increase in heart rate, peak ejection velocity, and S’, and a decrease in LVET and MVET, but preservation of LV end diastolic dimension, stroke volume, and eFS. There was no decrease in LV end systolic dimension, suggesting that although the rate of peak LV contraction is increased, this is counterbalanced by shorter ejection times, and therefore it does not increase stroke volumes. This more rapid ejection of a normal stroke volume could be caused by an increase in contractility due to sympathetic stimulation, circulating catecholamines, or the Treppe phenomenon (finding of contractility increasing with heart rate) [26]. Alternatively, because indices of ventricular performance such as eFS and S’ may be dependent on preload and afterload, alterations in LV loading conditions could be responsible. Examining the indices of preload and afterload, LVIDd and MRESS were similar in severe malaria to health, but SVRI and systolic blood pressure were reduced in malaria. When eFS was used as the measure of performance, adjusting for preload and afterload by regression [18], it was lower in uncomplicated malaria but not severe malaria than in healthy participants. When stroke power was used, which takes into account the reduced ejection time in malaria, this was higher in severe malaria cases than in healthy cases. Thus, when duration of contraction, preload, and afterload are taken into consideration, ventricular performance appears moderately elevated in severe malaria relative to healthy people at rest.

Within the severe malaria group, tachycardia, which was associated with fever but not with anemia, inversely correlated with LV dimensions and stroke volume. Anemia was associated with a larger fractional shortening and stroke volume, likely maintained by a more rapid ventricular ejection (LVOT_vmax_ and S’) and filling (larger E). These findings are in keeping with studies in children with malaria and anemia, where cardiac index has been found to increase [19], predominantly because of an increase in stroke volume [10]. After accounting for preload and afterload, hematocrit was not an independent predictor of eFS, suggesting that anemia leads to increased stroke volume predominantly by affecting preload and/or afterload as opposed to being associated with increased contractility. Consistent with this, hematocrit inversely correlated with vascular resistance as noted previously (H. W. F. K., manuscript in preparation), and on multivariate analysis anemia was associated with a larger LVIDd.

Hyperlactatemia, a consistent feature of severe malaria, was associated with tachycardia, a reduction in LV but not IVC dimensions, and an increase in eFS and a preserved SI, consistent with sympathetic stimulation causing a hyperdynamic state. Fatal cases had features consistent with relative hypovolemia and reduced cardiac index reserve, ie, smaller cardiac chamber volumes, increased relative wall thicknesses despite no increases in mass (pseudohypertrophy [27]), and more collapsible IVC. Patients with less cardiac index reserve may be unable to maintain or increase their cardiac index in response to stresses and would therefore be more likely to develop shock. Previous studies have shown that mortality in severe malaria increases with age, which is known to correlate with a decrease in cardiovascular reserve [28]. In healthy individuals, markers of myocardial relaxation deteriorate with aging [29]. This is consistent with the observation in this study that myocardial relaxation (E’ and IVRT) worsened significantly with age in the severe malaria group.

In bacterial sepsis, as in severe malaria, vascular resistance is typically reduced and the circulation is typically hyperdynamic (H. W. F. K., manuscript in preparation) [30]. Left ventricular systolic, diastolic, and RV function have been assessed in a wide range of ways in sepsis [31], although not typically in the context of preload and afterload. Where loading conditions have been considered, strong associations of contractility indices with afterload were observed in sepsis, confounding their interpretation in isolation [32]. Reduced indices of ventricular function have been reported more frequently in sepsis than we observed in severe malaria [33]. Patients described in studies of cardiac function in sepsis tend to be much older, with a mean age 50–60 [34] as opposed to median age 30 in this study, which may partially explain the differences in the frequency of abnormalities we observed. In addition, patients may have already received intensive care support including inotropic agents when the study began, which may have complicated assessment of contractility [35]. Left ventricular ejection fraction has not been consistently linked to mortality in sepsis, but smaller nonindexed LV dimensions have been linked in meta-analyses.

Using a noninvasive approach maximized recruitment into the study. However, this meant that chamber pressures and central venous oxygen saturations were not measured. In particular, in assessment of contractility and diastolic function, the assessment of pressure-volume loops would be informative. We have not assessed ventriculo-arterial coupling, a factor that can affect cardiac performance [36]. Adolescents were included alongside adults; severe malaria presents similarly in children over 10 years old as in adults [28]. Dimensions were adjusted for body size. Detailed studies were performed at only 1 of the study sites, and additional studies will be required to assess the generalizability of these findings in other settings. Inclusion of exercising or anemic healthy participants would be of value for controlling for the effects of heart rate and hematocrit.

## CONCLUSIONS

In conclusion, our study shows that impaired systolic function is typically absent in malaria and does not predict mortality. Diastolic LV function in malaria patients is usually not impaired and varies with age. Stroke volume index is maintained in adult patients with malaria despite a reduction in LV filling and ejection time. Patients who died from severe malaria had evidence of hypovolemia at baseline and evidence of reduced cardiac index reserve. Future studies should investigate whether tailoring fluid therapy to optimize volume status in these patients is beneficial.

## Supplementary Data

Supplementary materials are available at *The Journal of Infectious Diseases* online. Consisting of data provided by the authors to benefit the reader, the posted materials are not copyedited and are the sole responsibility of the authors, so questions or comments should be addressed to the corresponding author.

jiz568_suppl_Supplementary_Methods

jiz568_suppl_Supplementary_Tables

## Notes

Presented in part: American Society of Tropical Medicine and Hygiene, November 2017.

## Notes

***Acknowledgments.*** We are grateful for the patients’ consent to participate in these studies and to their relatives. We thank the doctors and staff involved in this project, including those of Chittagong Medical College and ISPAT General Hospital for support.

***Financial support.*** This work was funded by the Australian National Health and Medical Research Council (Grants 605807, 496600, and 1037304) and Fellowships (1042072; to N. M. A.), an Australian Government UPRS & PIRTS Scholarship (to H. W. F. K.), and the Radcliffe Travelling Fellowship of University College, Oxford (to M. T. H.). Mahidol Oxford Tropical Medicine Research Unit (MORU) is supported by the Wellcome Trust as part of the Wellcome Trust Major Overseas Programme.

***Potential conflicts of interest.*** All authors: No reported conflicts of interest. All authors have submitted the ICMJE Form for Disclosure of Potential Conflicts of Interest.
