# Supplementary material for: Reduced Cardiac Index Reserve and Hypovolemia in Severe Falciparum Malaria
Source: J Infect Dis. 2019 Nov 6;221(9):1518–27. doi: 10.1093/infdis/jiz568 (PMC7137886; doi:10.1093/infdis/jiz568)
Supplement: jiz568_suppl_Supplementary_Methods [file jiz568_suppl_supplementary_methods.docx]

**Supplemental methods**

**Patients**

CMCH is a 1000 bed tertiary referral hospital with 12 intensive care beds. IGH is a 685 bed hospital with 11 intensive care beds. In both hospitals, hemodialysis is available during office hours. Patients with malaria were treated according to local and international guidelines; patients with severe malaria received at least three doses of intravenous artesunate followed by 3 days of oral artemether-lumefantrine when able to take oral drugs (1). Patients with uncomplicated malaria received 3 days of oral artemether-lumefantrine. Most patients with severe disease were treated on the general ward because of limited intensive care bed capacity. Mortality was assessed in hospital. Arterial oxygen saturation was assessed by pulse oximetry and blood pressure was measured non-invasively. Mean arterial pressure (MAP) was estimated as 1/3 pulse pressure + diastolic blood pressure (DBP).

**Echocardiography**

Chamber volume measurements were made following the American society for echocardiography (ASE) guidelines (2). LV internal diameter at end systole (LVIDs) and diastole (LVIDd), posterior wall thickness (LVPWd) and septal thickness (IVSd) in diastole were measured from 2D parasternal long axis (PLAX) images at or just below the mitral valve tips. Left ventricular end diastolic volume index was estimated as the cube of LVIDd. Relative wall thickness was calculated as 2 times the LV posterior wall thickness divided by LVIDd. Left atrial diameter (LAd) was measured in the PLAX view. Proximal right ventricular outflow tract diameter (RVOTprox) was measured in the PLAX view. Inferior vena cava (IVC) diameter in expiration (IVCe) and inspiration (IVCi) were measured in the subcostal view. IVC collapsibility was calculated as (IVCe – IVCi)/IVCe. Tricuspid annular plane systolic excursion (TAPSE) was measured from peak to trough using M-mode at the tricuspid annulus from the apical four chamber view (A4C) (3). Right atrial (RA) area was measured from the A4C view. Early mitral peak E-wave velocity (E) was measured by pulse wave doppler in the A4C view following the ASE guidelines (4). Tissue Doppler measurements of the systolic (S’) and early diastolic (E’) medial and lateral mitral annular peak velocities were made from A4C views following the ASE guidelines (4). Medial and lateral values were averaged to give average S’ and average E’. LV Isovolaemic relaxation time (IVRT), LV ejection time (LVET) and mitral valve ejection time (MVET) were measured using the continuous wave (CW) doppler with the cursor positioned between the mitral and aortic valves (5). LVOT_Vmax_ and the aortic-velocity time integral (AVTI) were measured using pulsed wave (PW) Doppler from the apical 5 chamber (A5C) view. Stroke volume was estimated using the AVTI and the left ventricular outflow tract (LVOT) diameter from the PLAX view (6) by the equation: Stroke volume = AVTI.π(LVOT diameter/2)^2^. The LVOT diameter was measured at least three times and the maximum value obtained used. Cardiac output as calculated as the product of stroke volume and heart rate. Measurements were indexed to body surface area (BSA) (7). The systemic vascular resistance index (SVRI) was calculated as the ratio of MAP to cardiac index. Endocardial fractional shortening (eFS) was calculated as (LVIDd-LVIDs)/LVIDd and LV mass was estimated using the ASE recommended formula (2). Mediorotational end systolic stress (MRESS), a measure of LV afterload, was calculated from measurements of LVID, LVPWd and non-invasive systolic blood pressure (8). Stroke work index (SWI) was calculated as 0.0136 x stroke volume x MAP/BSA, stroke power index (SPI) as the ratio of stroke work index to ejection time and cardiac power index (CPI) as stroke work index multiplied by heart rate (9). Two cardiologists (VR and SS) supervised the analyses of all the echocardiographic data. All measurements were made in triplicate and the average values used for analysis. In 2011-2012 only cardiac index and IVC diameter were assessed and the assessments were performed by 4 trained doctors. From 2013 onwards the additional parameters described above were added and one clinician (HK) performed and analyzed all echocardiograms under the supervision of VR and SS.

**Supplemental figure 1. Enrolment flowchart**


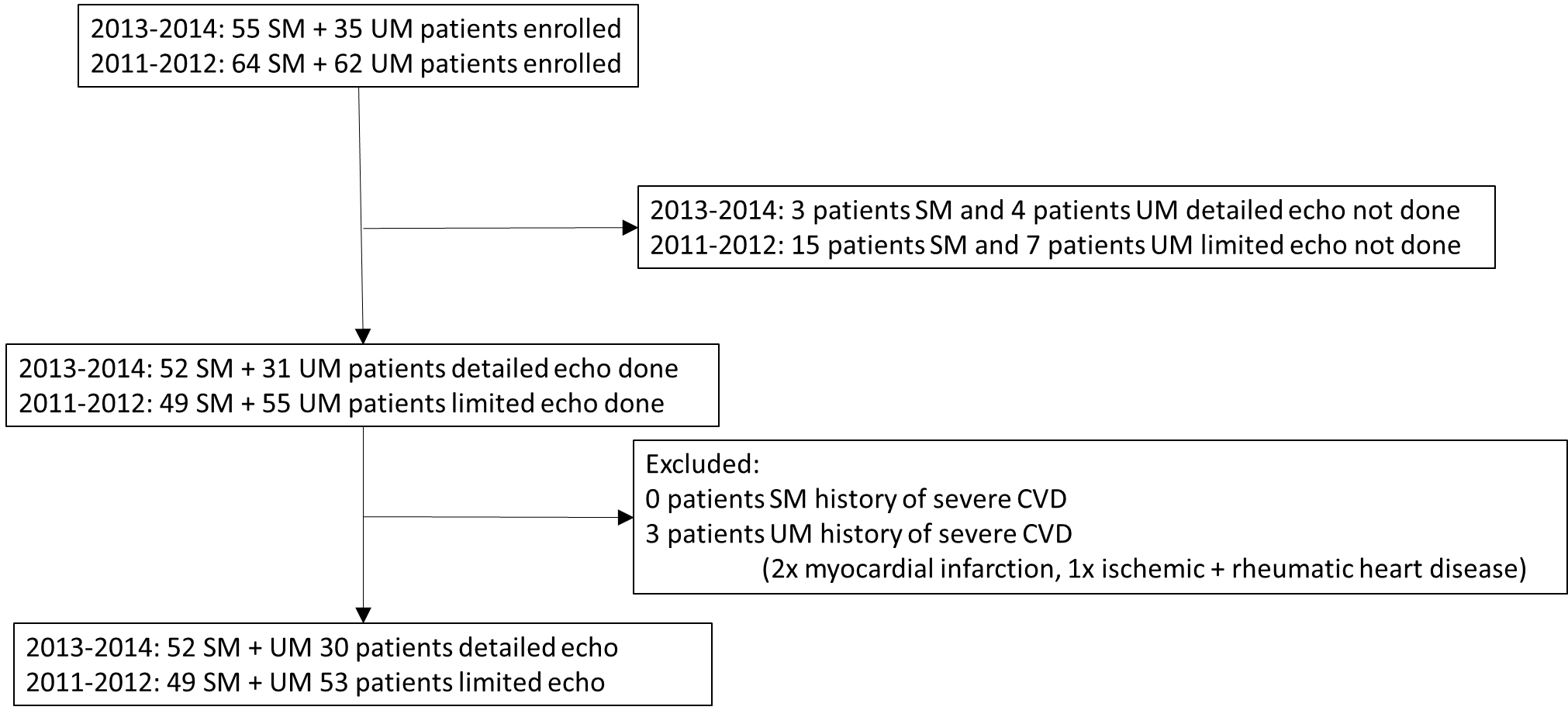


SM = severe malaria. UM = uncomplicated malaria. CVD = cardiovascular disease.

**References**

1. WHO. Guidelines for the treatment of malaria. 2015.

2. Lang RM, Bierig M, Devereux RB, et al. Recommendations for chamber quantification: a report from the American Society of Echocardiography's Guidelines and Standards Committee and the Chamber Quantification Writing Group, developed in conjunction with the European Association of Echocardiography, a branch of the European Society of Cardiology. Journal of the American Society of Echocardiography : official publication of the American Society of Echocardiography 2005;18(12):1440-1463.

3. Rudski LG, Lai WW, Afilalo J, et al. Guidelines for the echocardiographic assessment of the right heart in adults: a report from the American Society of Echocardiography endorsed by the European Association of Echocardiography, a registered branch of the European Society of Cardiology, and the Canadian Society of Echocardiography. Journal of the American Society of Echocardiography : official publication of the American Society of Echocardiography 2010;23(7):685-713; quiz 786-688.

4. Nagueh SF, Appleton CP, Gillebert TC, et al. Recommendations for the evaluation of left ventricular diastolic function by echocardiography. Journal of the American Society of Echocardiography : official publication of the American Society of Echocardiography 2009;22(2):107-133.

5. Quinones MA, Otto CM, Stoddard M, et al. Recommendations for quantification of Doppler echocardiography: a report from the Doppler Quantification Task Force of the Nomenclature and Standards Committee of the American Society of Echocardiography. Journal of the American Society of Echocardiography : official publication of the American Society of Echocardiography 2002;15(2):167-184.

6. Huntsman LL, Stewart DK, Barnes SR, et al. Noninvasive Doppler determination of cardiac output in man. Clinical validation. Circulation 1983;67(3):593-602.

7. Bansal M, Mohan JC, Sengupta SP. Normal echocardiographic measurements in Indian adults: How different are we from the western populations? A pilot study. Indian heart journal 2016;68(6):772-775.

8. de Simone G, Devereux RB, Roman MJ, et al. Assessment of left ventricular function by the midwall fractional shortening/end-systolic stress relation in human hypertension. J Am Coll Cardiol 1994;23(6):1444-1451.

9. Varon J, Fromm RE. Cardiovascular Facts and Formulas. In: Acute and Critical Care Formulas and Laboratory Values. New York, NY: Springer New York; 2014. p. 1-23.
