# Supplementary material for: Reduced Cardiac Index Reserve and Hypovolemia in Severe Falciparum Malaria
Source: J Infect Dis. 2019 Nov 6;221(9):1518–27. doi: 10.1093/infdis/jiz568 (PMC7137886; doi:10.1093/infdis/jiz568)
Supplement: jiz568_suppl_Supplementary_Tables [file jiz568_suppl_supplementary_tables.docx]

**Supplementary table 1.**

|  | SI (ml/m^2^) | CI (ml/^2^) | SVRI (dynes.sec/cm^5^/m^2^) | SWI (g.m/m^2^) | SPI (W/m^2^) | CPI (W/m^2^) |
| --- | --- | --- | --- | --- | --- | --- |
| Measurements |  |  |  |  |  |  |
| Healthy | 38.3 (33.2 to 43.8) N=43 | 2726 (2340 to 3100) N=43 | 2789 (2457 to 3293) N=43 | 45.84 (39.58 to 52.8) N=43 | 1.57 (1.4 to 1.74) N=28 | 0.54 (0.46 to 0.59) N=43 |
| Uncomplicated | 40.1 (35.1 to 46.6) N=80 | 3791 (3173 to 4439) N=79 | 1647 (1375 to 2206) N=79 | 41.45 (37.24 to 49.45) N=80 | 1.73 (1.46 to 2.14) N=27 | 0.66 (0.54 to 0.78) N=79 |
| Severe | 38.3 (30.8 to 45.6) N=94 | 3997 (3288 to 4818) N=94 | 1658 (1291 to 2094) N=94 | 42.09 (34.91 to 52.15) N=94 | 1.78 (1.56 to 2.17) N=45 | 0.73 (0.6 to 0.86) N=94 |
| Severe - Alive | 39.6 (32 to 45.6) N=66 | 3912 (3360 to 4742) N=66 | 1616 (1338 to 2093) N=66 | 43.49 (35.53 to 52.15) N=66 | 1.7 (1.48 to 2.03) N=30 | 0.71 (0.57 to 0.84) N=66 |
| Severe - Dead | 34.7 (26.6 to 45.5) N=28 | 4056 (3095 to 4880) N=28 | 1671 (1117 to 2301) N=28 | 36.23 (33.35 to 52.02) N=28 | 1.91 (1.57 to 2.42) N=15 | 0.73 (0.64 to 0.89) N=28 |
| P values |  |  |  |  |  |  |
| Overall (Kruskal-Wallis) | 0.282 | <0.001 | <0.001 | 0.061 | 0.042 | <0.001 |
| Healthy vs Uncomplicated | 0.205 | <0.001 | <0.001 | 0.032 | 0.043 | <0.001 |
| Healthy vs severe | 0.889 | <0.001 | <0.001 | 0.032 | 0.019 | <0.001 |
| Uncomplicated vs severe | 0.155 | 0.332 | 0.585 | 0.742 | 0.758 | 0.039 |
| Alive vs dead | 0.155 | 0.679 | 0.779 | 0.282 | 0.373 | 0.423 |

SI = stroke index; CI = cardiac index; SWI = stroke work index; SPI = stroke power index; CPI = cardiac power index. Shown is median (interquartile range)

**Supplementary table 2. Chamber volumes**

|  | LVIDd/BSA (cm/m^2^) | LVIDs/BSA (cm/m^2^) | LV Mass (ASE)/BSA (g/m^2^) | RWT | LAd/BSA (cm/m^2^) | RVOTprox/BSA (cm/m^2^) | RA area/BSA (cm^2^/m^2^) | IVCc | IVCe (cm) | IVCi(cm) |
| --- | --- | --- | --- | --- | --- | --- | --- | --- | --- | --- |
| ASE Normal ranges |  |  |  |  |  |  |  |  |  |  |
| Female | 2.2–3.0 | 1.3–2.1 | 49–115 | 0.22-0.42 | 1.5–2.3 |  |  |  |  |  |
| Male | 2.3–3.1 | 1.3–2.1 | 43–95 | 0.22-0.42 | 1.5–2.3 |  |  |  |  |  |
| Measurements |  |  |  |  |  |  |  |  |  |  |
| Healthy | 2.7 (2.4 to 2.8) N=28 | 1.8 (1.5 to 1.9) N=28 | 75.6 (66.9 to 86) N=28 | 0.39 (0.35 to 0.42) N=28 | 1.6 (1.4 to 1.8) N=28 | 1.7 (1.5 to 1.8) N=27 | 6.8 (5.7 to 7.3) N=26 | 0.4 (0.2 to 0.5) N=41 | 1.3 (1.1 to 1.5) N=41 | 0.8 (0.6 to 1.1) N=41 |
| Uncomplicated | 2.9 (2.7 to 3.2) N=30 | 2 (1.7 to 2.2) N=30 | 83 (70.7 to 100.9) N=30 | 0.41 (0.36 to 0.43) N=30 | 1.8 (1.6 to 2) N=30 | 1.7 (1.6 to 1.9) N=30 | 7.1 (5.7 to 8.7) N=21 | 0.5 (0.2 to 0.8) N=75 | 1.2 (0.9 to 1.5) N=77 | 0.6 (0.2 to 1) N=75 |
| Severe | 2.8 (2.5 to 3.1) N=52 | 1.9 (1.6 to 2.2) N=52 | 81.2 (67.8 to 94.6) N=52 | 0.39 (0.35 to 0.47) N=52 | 1.6 (1.4 to 1.8) N=52 | 1.6 (1.5 to 1.8) N=52 | 6.1 (5.2 to 7) N=45 | 0.5 (0.3 to 0.8) N=86 | 1.1 (0.8 to 1.5) N=87 | 0.4 (0.1 to 1) N=86 |
| Severe - Alive | 2.9 (2.7 to 3.2) N=33 | 2 (1.7 to 2.2) N=33 | 86.4 (72.1 to 93.8) N=33 | 0.36 (0.35 to 0.43) N=33 | 1.7 (1.4 to 1.8) N=33 | 1.6 (1.5 to 1.8) N=33 | 6.5 (5.7 to 7.8) N=30 | 0.5 (0.3 to 0.8) N=61 | 1.2 (0.8 to 1.5) N=62 | 0.6 (0.3 to 1.1) N=61 |
| Severe - Dead | 2.6 (2.2 to 2.8) N=19 | 1.6 (1.4 to 1.9) N=19 | 72.8 (66.1 to 96.3) N=19 | 0.46 (0.36 to 0.64) N=19 | 1.6 (1.2 to 1.9) N=19 | 1.6 (1.4 to 1.8) N=19 | 5.2 (4.1 to 6.8) N=15 | 0.7 (0.5 to 0.9) N=25 | 1.1 (0.7 to 1.3) N=25 | 0.3 (0.1 to 0.5) N=25 |
| P values |  |  |  |  |  |  |  |  |  |  |
| Overall (Kruskal-Wallis) | 0.035 | 0.075 | 0.167 | 0.584 | 0.017 | 0.564 | 0.051 | 0.011 | 0.025 | 0.004 |
| Healthy vs Uncomplicated | 0.008 | 0.026 | 0.081 | 0.29 | 0.02 | 0.424 | 0.207 | 0.037 | 0.092 | 0.02 |
| Healthy vs severe | 0.069 | 0.113 | 0.13 | 0.408 | 0.687 | 0.772 | 0.181 | 0.002 | 0.009 | 0.001 |
| Uncomplicated vs severe | 0.381 | 0.331 | 0.525 | 0.969 | 0.008 | 0.317 | 0.022 | 0.405 | 0.173 | 0.335 |
| Alive vs dead | 0.006 | 0.003 | 0.093 | 0.037 | 0.562 | 0.442 | 0.025 | 0.006 | 0.423 | 0.014 |

BSA = body surface area; LVIDd = left ventricular (LV) internal dimension in diastole; LVIDs = LV internal dimension in systole; ASE = American Society for Echocardiography; PSAX = parasternal short axis; RVOTprox = proximal right ventricular outflow tract; RA = right atrium; IVCc = inferior vena cava (IVC) collapsibility index; IVCe = IVC expiratory diameter. IVCi = IVC inspiratory diameter. Shown is median (interquartile range)

**Supplementary table 3. Systolic variables**

|  | Endocardial FS (%) | Average S' (cm/s) | LVOT_vmax_ (m/s) | LVET (ms) | MRESS (10^3^ dyne/cm^2^) | TAPSE (cm) |
| --- | --- | --- | --- | --- | --- | --- |
| ASE Normal ranges |  |  |  |  |  |  |
| Female | 25–43 |  |  |  |  |  |
| Male | 27–45 |  |  |  |  |  |
| Measurements |  |  |  |  |  |  |
| Healthy | 33.8 (30.7 to 37.4) N=28 | 9 (8.1 to 9.9) N=28 | 1.02 (0.96 to 1.13) N=30 | 272 (251 to 287) N=29 | 61 (48 to 67) N=28 | 2 (1.8 to 2.1) N=26 |
| Uncomplicated | 31.7 (27.9 to 37.1) N=30 | 10.8 (9.2 to 12) N=29 | 1.29 (1.12 to 1.4) N=30 | 229 (212 to 251) N=28 | 55 (47 to 63) N=30 | 2.3 (2.1 to 2.5) N=29 |
| Severe | 32.2 (27.3 to 39.1) N=52 | 11.8 (10 to 13.6) N=51 | 1.17 (1.04 to 1.39) N=52 | 205 (170 to 230) N=50 | 52 (39 to 69) N=52 | 2 (1.8 to 2.5) N=49 |
| Severe - Alive | 31.3 (26.4 to 35.8) N=33 | 11.8 (10.1 to 12.4) N=32 | 1.17 (1.01 to 1.35) N=33 | 214 (194 to 241) N=31 | 57 (44 to 75) N=33 | 2.1 (1.9 to 2.5) N=32 |
| Severe - Dead | 36.9 (28.2 to 46.9) N=19 | 12.9 (9.2 to 15.7) N=19 | 1.14 (1.07 to 1.5) N=19 | 176 (169 to 205) N=19 | 49 (26 to 56) N=19 | 2 (1.6 to 2.4) N=17 |
| P values |  |  |  |  |  |  |
| Overall (Kruskal-Wallis) | 0.469 | <0.001 | <0.001 | <0.001 | 0.59 | 0.042 |
| Healthy vs Uncomplicated | 0.181 | 0.002 | <0.001 | <0.001 | 0.401 | 0.005 |
| Healthy vs severe | 0.525 | <0.001 | 0.001 | <0.001 | 0.369 | 0.672 |
| Uncomplicated vs severe | 0.538 | 0.163 | 0.096 | 0.001 | 0.672 | 0.081 |
| Alive vs dead | 0.093 | 0.134 | 0.464 | 0.014 | 0.037 | 0.179 |
|  |  |  |  |  |  |  |
|  | | | | | | |

FS = fraction shortening; LVOT_vmax_ = peak Left ventricular outflow tract velocity; TAPSE = tricuspid annular plane systolic excursion; LVET = Left ventricular ejection time; MRESS = mediorotational end systolic stress. Shown is median (interquartile range)

**Supplementary table 4. Linear regression models for eFS, SWI and SPI**

| Dependent variable |  | LVIDd (cm) | MRESS (x10^3^ dynes/cm^2^) | Sex (male) | UM | SM | Constant | R^2^ |
| --- | --- | --- | --- | --- | --- | --- | --- | --- |
| eFS (%) |  | 3.7 (1.4 to 5.9) | -0.31 (-0.37 to -0.26) | -2.2 (-4.3 to -0.1) | -3.6 (-6 to -1.2) | -1.7 (-3.9 to 0.5) | 38 (29 to 47) | 0.62 |
|  |  |  |  |  |  |  |  |  |
|  |  | LVEDVI (ml^3^/m^2^) |  | Sex (male) | UM | SM | Constant | R^2^ |
| SWI (g.m/m^2^) |  | 0.41 (0.29 to 0.53) |  | -1.71 (-5.71 to 2.29) | -2.89 (-7.68 to 1.9) | -4.9 (-9.08 to -0.71) | 23.4 (15.9 to 31) | 0.36 |
|  |  |  |  |  |  |  |  |  |
|  |  | LVEDVI (ml^3^/m^2^) |  | Sex (male) | UM | SM | Constant | R^2^ |
| SPI (W/m^2^) |  | 0.011 (0.005 to 0.017) |  | -0.04 (-0.24 to 0.16) | 0.21 (-0.03 to 0.45) | 0.29 (0.08 to 0.5) | 1.02 (0.63 to 1.4) | 0.19 |
|  |  |  |  |  |  |  |  |  |
|  | | | | | | | | |
|  |  |  |  |  |  |  |  |  |

Shown are linear regression coefficients with 95% confidence intervals. eFS = endocardial fractional shortening, SWI = Stroke work index, SPI = LV stroke power index, LVIDd = LV internal diameter end diastole, LVEDVI = LV end diastolic volume index, MRESS = mediorotational end systolic stress, UM = uncomplicated malaria, SM = severe malaria.

**Supplementary table 5. Diastolic variables**

|  | E velocity (m/s) | Average E' (cm/s) | E/E' (average) | IVRT (ms) | MVET (ms) |
| --- | --- | --- | --- | --- | --- |
| Measurements |  |  |  |  |  |
| Healthy | 0.8 (0.7 to 0.9) N=28 | 12 (10 to 13.5) N=28 | 6.7 (5.9 to 7.6) N=28 | 83.2 (73.9 to 91) N=29 | 435.3 (346.3 to 494.8) N=28 |
| Uncomplicated | 0.9 (0.8 to 1.1) N=30 | 13.4 (10.4 to 15.3) N=30 | 7.6 (6.4 to 8.3) N=30 | 58.5 (48.9 to 67.2) N=28 | 279.1 (244.6 to 333.3) N=30 |
| Severe | 0.9 (0.7 to 1) N=52 | 12.7 (9.7 to 15.2) N=51 | 7.2 (5.7 to 9) N=51 | 61.5 (52.5 to 76.9) N=48 | 270.5 (210.7 to 317.9) N=51 |
| Severe - Alive | 0.9 (0.7 to 1) N=33 | 13.2 (10.9 to 15.4) N=32 | 6.7 (5.9 to 8.2) N=32 | 61.3 (51.1 to 78.9) N=31 | 277.3 (228 to 340.1) N=33 |
| Severe - Dead | 0.9 (0.6 to 1.1) N=19 | 11 (7.8 to 15.2) N=19 | 7.9 (5.7 to 9.7) N=19 | 66.5 (52.7 to 73.9) N=17 | 238.9 (182.3 to 297.6) N=18 |
| P values |  |  |  |  |  |
| Overall (Kruskal-Wallis) | 0.17 | 0.538 | 0.469 | <0.001 | <0.001 |
| Healthy vs Uncomplicated | 0.058 | 0.219 | 0.171 | <0.001 | <0.001 |
| Healthy vs severe | 0.175 | 0.573 | 0.532 | <0.001 | <0.001 |
| Uncomplicated vs severe | 0.531 | 0.591 | 0.564 | 0.175 | 0.278 |
| Alive vs dead | 0.932 | 0.205 | 0.459 | 0.94 | 0.037 |

IVRT = Isovolaemic relaxation time, MVET = mitral valve ejection time. Shown is median (interquartile range)

|  | Base deficit | GCS | IVCi | N | R^2^ |
| --- | --- | --- | --- | --- | --- |
| Model 1 | 0.84 (0.75 to 0.93), p = 0.001 | 0.81 (0.67 to 0.97), p = 0.02 | 0.23 (0.06 to 0.93), p = 0.039 | 86 | 0.28 |
|  |  |  |  |  |  |
|  | Base deficit | GCS | IVCc | N | R^2^ |
| Model 2 | 0.84 (0.75 to 0.93), p = 0.001 | 0.8 (0.67 to 0.96), p = 0.019 | 10.08 (1.28 to 79.16), p = 0.028 | 86 | 0.29 |
|  |  |  |  |  |  |
|  | Base deficit | GCS | LVIDs | N | R^2^ |
| Model 3 | 0.88 (0.77 to 1.01), p = 0.08 | 0.6 (0.42 to 0.84), p = 0.003 | 0.04 (0 to 0.55), p = 0.017 | 52 | 0.45 |
|  |  |  |  |  |  |
|  | Base deficit | GCS | Heart rate | N | R^2^ |
| Model 4 | 0.89 (0.81 to 0.97), p=0.009 | 0.77 (0.65 to 0.9), p=0.001 | 1.02 (1 to 1.05), p=0.041 | 101 | 0.25 |
|  |  |  |  |  |  |

**Supplementary table 6. Prediction of outcome in severe malaria by logistic regression**

Logistic regression models for outcome in severe malaria. Shown is odds ratio and 95% confidence interval. GCS = Glasgow coma score, IVCi = inferior vena cava diameter on inspiration, IVCi = inferior vena cava collapsibility, LVIDs = Left ventricular internal diameter in systole

**Supplementary table 7. List of cardiac variables**

|  | Abbreviation | View | Notes |
| --- | --- | --- | --- |
| Left ventricular internal diameter in diastole | LVIDd | PLAX | Internal diameter of the LV cavity prior to contraction. |
| Left ventricular internal diameter in systole | LVIDs | PLAX | Internal diameter of the LV cavity prior after contraction. |
| Left ventricular mass | LV mass |  | Estimated LV muscle mass; increased in LV hypertrophy [1]. |
| Relative wall thickness of the left ventricle | RWT |  | Thickness of LV walls relative to cavity; increases in LV hypertrophy, hypovolemia, remodeling [1]. |
| Left atrial anterior-posterior dimension | LAd | PLAX | Size of LA influenced by compliance, filling time and pressure. |
| Right ventricular outflow tract proximal diameter | RVOTprox | PLAX | Related to RV size. |
| Right atrial area | RA area | A4C | Size of RA influenced by compliance, filling time and pressure. |
| Inferior vena cava expiratory diameter | IVCe | Subcostal | A correlate of CVP [2]. |
| Inferior vena cava Inspiratory diameter | IVCi | Subcostal |  |
| Inferior vena cava collapsibility | IVCc | Subcostal | Associated with fluid responsiveness [3]. |
| Mediorotational end systolic stress | MRESS |  | A measure of LV afterload [4]. |
| Endocardial fraction shortening | eFS |  | A measure of LV systolic function, not independent of preload and afterload. |
| Average mitral annular peak systolic tissue velocity | Average S' | A4C | Peak velocity of the mitral annulus during systole, a measure of LV systolic function [5]. Not independent of preload and afterload. |
| Left ventricular outflow tract maximum velocity | LVOTvmax | A5C | Peak LVOT velocity, a measure of LV systolic function [5]. Not independent of preload and afterload. |
| Tricuspid annular plane systolic excursion | TAPSE | A4C | A measure of RV systolic function [6]. |
| Left ventricular ejection time | LVET | A5C |  |
| Stroke index | SI |  | Stroke volume indexed to body surface area. |
| Cardiac index | CI |  | Stroke index multiplied by heart rate. |
| Stroke work index | SWI |  | A measure of the external work done by the heart per beat. |
| Stroke power index | SPI |  | Stroke work divided by ejection time. A measure of the rate at which external work is done by the heart per beat. |
| Cardiac power index | CPI |  | Stroke work index multiplied by heart rate. A measure of the rate at which external work is done by the heart [7]. |
| Early mitral peak E-wave velocity | E | A4C | Reflects the pressure gradient between LA and LV. Increases with increasing LA pressure and LV relaxation [8]. |
| Average of medial and lateral mitral annular peak early diastolic tissue velocity | Average E' | A4C | Increases with increasing LV relaxation rate and to a lesser extent LA pressure [8]. |
| Ratio of mitral early peak E-wave velocity to average mitral annular peak early diastolic tissue velocity | E/E' | A4C | Used as an index of LV filling pressure [8]. |
| Isovolaemic relaxation time | IVRT | A5C | Time between AV closure and MV opening [8]. Decreases with increasing LA pressure and LV relaxation rate. |
| Mitral valve ejection time | MVET | A5C | Duration of LV filling. |

1. Lang RM, Bierig M, Devereux RB, et al. Recommendations for chamber quantification: a report from the American Society of Echocardiography's Guidelines and Standards Committee and the Chamber Quantification Writing Group, developed in conjunction with the European Association of Echocardiography, a branch of the European Society of Cardiology. Journal of the American Society of Echocardiography : official publication of the American Society of Echocardiography **2005**; 18:1440-63.

2. Prekker ME, Scott NL, Hart D, Sprenkle MD, Leatherman JW. Point-of-care ultrasound to estimate central venous pressure: a comparison of three techniques. Crit Care Med **2013**; 41:833-41.

3. Preau S, Bortolotti P, Colling D, et al. Diagnostic Accuracy of the Inferior Vena Cava Collapsibility to Predict Fluid Responsiveness in Spontaneously Breathing Patients With Sepsis and Acute Circulatory Failure. Crit Care Med **2017**; 45:e290-e7.

4. de Simone G, Devereux RB, Roman MJ, et al. Assessment of left ventricular function by the midwall fractional shortening/end-systolic stress relation in human hypertension. J Am Coll Cardiol **1994**; 23:1444-51.

5. Thorstensen A, Dalen H, Amundsen BH, Stoylen A. Peak systolic velocity indices are more sensitive than end-systolic indices in detecting contraction changes assessed by echocardiography in young healthy humans. European journal of echocardiography : the journal of the Working Group on Echocardiography of the European Society of Cardiology **2011**; 12:924-30.

6. Rudski LG, Lai WW, Afilalo J, et al. Guidelines for the echocardiographic assessment of the right heart in adults: a report from the American Society of Echocardiography endorsed by the European Association of Echocardiography, a registered branch of the European Society of Cardiology, and the Canadian Society of Echocardiography. Journal of the American Society of Echocardiography : official publication of the American Society of Echocardiography **2010**; 23:685-713; quiz 86-8.

7. Fincke R, Hochman JS, Lowe AM, et al. Cardiac power is the strongest hemodynamic correlate of mortality in cardiogenic shock: a report from the SHOCK trial registry. J Am Coll Cardiol **2004**; 44:340-8.

8. Nagueh SF, Smiseth OA, Appleton CP, et al. Recommendations for the Evaluation of Left Ventricular Diastolic Function by Echocardiography: An Update from the American Society of Echocardiography and the European Association of Cardiovascular Imaging. Journal of the American Society of Echocardiography : official publication of the American Society of Echocardiography **2016**; 29:277-314.
